# Historical biogeography of the widespread macroalga *Sargassum* (Fucales, Phaeophyceae)

**DOI:** 10.1111/jpy.12945

**Published:** 2019-12-15

**Authors:** Zhi Ting Yip, Randolph Z. B. Quek, Danwei Huang

**Affiliations:** ^1^ Department of Biological Sciences National University of Singapore Singapore City 117558 Singapore; ^2^ Tropical Marine Science Institute National University of Singapore Singapore City 119227 Singapore

**Keywords:** ancestral range, brown macroalgae, cladogenesis, global distribution, macroevolution, time‐calibrated phylogeny

## Abstract

*Sargassum* is a cosmopolitan brown algal genus spanning the three ocean basins of the Atlantic, Pacific and Indian Oceans, inhabiting temperate, subtropical and tropical habitats. *Sargassum* has been postulated to have originated in the Oligocene epoch approximately 30 mya according to a broad phylogenetic analysis of brown macroalgae, but its diversification to become one of the most widespread and speciose macroalgal genera remains unclear. Here, we present a Bayesian molecular clock study, which analyzed data from the order Fucales of the brown algal crown radiation (BACR) group to reconstruct a time‐calibrated phylogeny of the *Sargassum* clade. Our phylogeny included a total of 120 taxa with 99 *Sargassum* species sampled for three molecular markers – *ITS‐2*,* cox3* and *rbc*LS – calibrated with an unambiguous Sargassaceae fossil from between the lower and middle Miocene. The analysis revealed a much later origin of *Sargassum* than expected at about 6.7 mya, with the genus diversifying since approximately 4.3 mya. Current geographic distributions of *Sargassum* species were then analyzed in conjunction with the time‐calibrated phylogeny using the dispersal‐extinction‐cladogenesis (DEC) model to estimate ancestral ranges of clades in the genus. Results strongly support origination of *Sargassum* in the Central Indo‐Pacific (CIP) region with subsequent independent dispersal events into other marine realms. The longer history of diversification in the ancestral CIP range could explain the much greater diversity there relative to other marine areas today. Analyses of these dynamic processes, when fine‐tuned to a higher spatial resolution, enable the identification of evolutionary hotspots and provide insights into long‐term dispersal patterns.

**Abbreviations**
BACR: brown algal crown radiation
*cox3*: mitochondrial cytochrome coxidase subunit 3
DEC: dispersal‐extinction‐cladogenesis
HPD: highest posterior density
*ITS‐2*: internal transcribed spacer 2 region of nuclear ribosomal RNA
MCC: maximum clade credibility
PP: posterior probability
*rbc*LS: chloroplastic ribulose bisphosphate carboxylase large subunit

Species richness is high in many marine environments despite the scarcity of hard barriers isolating populations from gene flow (Cowman and Bellwood 2013). While there is strong interest in the study of diversification and evolution of marine organisms (Vieira et al. 2017), the interconnectedness of the oceans makes it challenging to study the macroevolutionary processes driving the distribution of species (Cowman and Bellwood 2013). Fundamentally, spatial variation in species richness over evolutionary timescales can be explained by biogeographic models, which make predictions about the relative contributions of the primary macroevolutionary processes, speciation, extinction and dispersal (Stebbins 1974, Jablonski 1993, Jablonski et al. 2006). Interestingly, marine taxa exhibiting concordant diversity distributions may have discordant and complex biogeographic histories (Bowen et al. 2013). For example, the high species diversity of reef fish in the Indo‐West Pacific region has been shown to be driven by rapid speciation (Mora et al. 2003, Tornabene et al. 2015) while the high coral diversity could have resulted from long‐term dispersal of species into this region (Connolly et al. 2003, Huang et al. 2018).

While many marine groups, including corals, cowries and shore fishes, exhibit high relative diversity in the tropical Indo‐West Pacific region (Hughes et al. 2002, Bellwood et al. 2005, 2012, Bellwood and Meyer 2009), marine algae are typically more genus rich in temperate regions (Kerswell 2006). Despite this, there are taxon‐specific patterns where high algal richness has been observed in the tropics, specifically in the central Indo‐Pacific region such as for the brown macroalga *Lobophora* (Dictyotales, Phaeophyceae; Vieira et al. 2017) and red macroalga *Portieria* (Gigartinales, Rhizophyllidaceae; Leliaert et al. 2018). Likewise, the cosmopolitan genus *Sargassum*, which comprises over 350 currently accepted species (Guiry and Guiry 2018), exhibits a similar pattern by dominating the tropical and subtropical regions (Mattio and Payri 2011). It is also especially species rich in the Pacific (Phillips 1995), although few studies on marine algae, apart from *Fucus* (Cánovas et al. 2011) and *Lobophora* (Vieira et al. 2017), have tested biogeographic hypotheses of the richness divergence between the Pacific and Atlantic Oceans. Despite the widespread and far‐ranging ecological associations *Sargassum* has with other marine organisms – from being a food source and providing key tropical habitats equivalent to the temperate kelp forests (Mattio and Payri 2011, Mattio et al. 2013), to being a potent competitor of corals on reef environments (McManus and Polsenberg 2004) – the contemporary distribution and historical biogeography of the genus remain poorly understood.

Recent availability of DNA sequence data for a large number of *Sargassum* species has improved our understanding of phylogenetic relationships within the group (Mattio et al. 2008, 2009, 2015, Mattio and Payri 2009, Yip et al. 2018). When calibrated using relevant paleontological data, the phylogeny can be used to infer species ancestral ranges and the origination of the genus (Moore and Donoghue 2007). Paleontological data on brown macroalgae (Phaeophyceae), such as *Sargassum*, are sparse due to their general inability to fossilize (Silberfeld et al. 2010). Nevertheless, the diversification of *Sargassum* has been estimated to be relatively recent – no earlier than the Neogene period (Silberfeld et al. 2010). Therefore, like for many successful marine taxa, late geological events in the Pliocene may have been the main drivers of its diversification and distribution (Hallam 1985).

Biogeographic studies of brown macroalgal groups have focused on the macroevolution of common and widespread genera like *Macrocystis* (Rothman et al. 2017), *Lobophora* (Vieira et al. 2017), and *Fucus* (Cánovas et al. 2011). Those of *Sargassum* are largely limited to the use of hydrodynamic and ocean current models. For example, Mattio et al. (2015) utilized hydrodynamic models to characterize dispersal patterns between isolated islands of the western Indian Ocean, while Phillips (1995) invoked ocean circulation to explain why the western Pacific is more diverse than eastern Pacific despite higher endemism in the latter. By leveraging on a more robust understanding of the *Sargassum* phylogeny, biogeographic analyses of species ancestral ranges could be performed to reconstruct macroevolutionary processes that have shaped present species distributions and diversity gradients.

Congruent diversity patterns between macroalgae and other major coral reef taxa such as fishes and corals have generally been assumed to result from associations that drive the diversification of reef organisms as a whole (Hughes et al. 2002, Cowman and Bellwood 2011). For example, ecological interactions like herbivory have been known to play key roles in macroalgal evolution (Hay 1997), suggesting that diversification trajectories of macroalgae and their herbivores may be correlated. Furthermore, as the foundation of reef habitats, corals provide opportunities for new niche colonization that may promote reef algal speciation (Cowman and Bellwood 2011). As such, the importance of investigating diversity and distribution patterns of *Sargassum* are underlain by its interactions with modern corals. Macroalgae compete directly with hard corals via several means, including shading, physical abrasion, competition for space, and impacting coral larval settlement (McCook et al. 2001). Thus, phase shift to macroalgal dominance by this ubiquitous reef genus is a serious threat to corals and an indication of poor reef health (Azevedo et al. 2011, Smith et al. 2016).

Apart from co‐diversification of reef organisms, there are other hypotheses and growing evidence suggesting that large‐scale geological events and tectonic activities could account for concordant biogeographic processes and diversification patterns, particularly between reef fishes and corals (Keith et al. 2013, Leprieur et al. 2016). In addition, availability of habitats and empty niches promotes speciation, which could also result in similar patterns across taxa (Moura et al. 2013, Sanciangco et al. 2013). Therefore, understanding the processes that drive diversification and diversity patterns involves integrating evolutionary models with paleoclimatic and paleoceanographic data. In uncovering the macroevolutionary patterns of *Sargassum* that have led to the contemporary global distribution and species richness gradients (Okamura 1932, Yoshida 1989, Phillips 1995), biodiversity drivers on habitats in which *Sargassum* thrives could eventually be elucidated (Etti and Schils 2016).

In this study, we inferred the most comprehensive time‐calibrated phylogeny of *Sargassum* yet, based on all available sequences of three commonly analyzed markers to estimate the age and origin of *Sargassum*. Species distribution data were then consolidated from isolated diversity studies over the last decade (Mattio et al. 2008, 2009, 2015, Mattio and Payri 2009, Nguyen 2014) and analyzed in conjunction with the phylogeny using a biogeographic model to estimate ancestral ranges and macroevolutionary processes (dispersal‐extinction‐cladogenesis; Ree and Smith 2008). Our reconstruction supports a Central Indo‐Pacific origination and initial diversification of *Sargassum* with very recent (<1.5 mya) independent dispersals into the Atlantic, resulting in high species richness in the Pacific but limited diversity and endemism in the Atlantic.

## Materials and Methods

### Taxon sampling and alignment

Molecular sequences of nuclear *ITS‐2*, chloroplastic partial RuBisCO operon *rbc*LS, and mitochondrial *cox*3 for *Sargassum* species were downloaded from the NCBI GenBank database (Table S1 in the Supporting Information). Sequences were selected based on a set of criteria to maximize taxonomic accuracy. Specifically, we preferred sequences generated in the recent *Sargassum*‐focused taxonomic work by Mattio et al. (2008, 2009, 2010, 2013, 2015, 2010), Mattio and Payri (2009), followed by sequences from other published studies (Camacho et al. 2015) and, lastly, unpublished sequences uploaded by taxonomists. After this screening process, data were found to be available for 99 species, representing 28% of all valid *Sargassum* species (Guiry and Guiry 2018). Gene sequences used for each species may not have been sampled from the same individual so as to increase gene coverage and reduce missing data. Sequences from Silberfeld et al. (2010) representing outgroup species from the Fucales order and *Turbinaria* sequences from Stiger et al. (2003) were also downloaded and included in the analysis. The 120‐taxon data matrices were each aligned with MAFFT 7.311 (Katoh and Standley 2013) using the L‐INS‐I alignment algorithm and trimmed to 710, 434, and 755 sites for *ITS‐2*,* cox*3, and *rbc*LS respectively in Mesquite 3.2 (Maddison and Maddison 2017), before being concatenated for phylogenetic analyses.

### Bayesian divergence time‐estimated phylogeny

The best‐fit substitution model for each gene alignment was estimated using jModelTest2 (Guindon and Gascuel 2003, Darriba et al. 2012), comparing the fit of various models under the Akaike information criterion (*ITS‐2*: GTR + Γ; *cox3* and *rbc*LS: GTR + I + Γ). Substitution models were included as priors in a Bayesian analysis to infer the time‐calibrated phylogeny of Fucales focusing on the genus *Sargassum* using a relaxed molecular clock implemented in BEAST 2.4.6 (Huelsenbeck et al. 2001, Drummond and Rambaut 2007, Bouckaert et al. 2014). The three gene partitions were linked with an uncorrelated lognormal clock model and a birth–death tree prior.

The lack of a continuous and well‐characterized fossil record stemmed from the nature of brown algal groups, which comprised only soft tissues (Silberfeld et al. 2010). The only geological formation that yielded fossils for calibrating the Fucales phylogeny was associated with the Sargassaceae family. The monophyletic group of the order Fucales, excluding the deepest branch *Notheia anomala* (Notheiceae), was the only node used as a calibration point by applying Sargassaceae fossils previously implemented in Silberfeld et al. (2010). The structurally complex fossils found in the Miocene deposits (13–17 mya) of the Monterey formation (Parker and Dawson 1965) belonged to the extinct genera *Paleocystophora* (*P. subopposita*) and *Paleohalidrys (P. californica, P. superba,* and *P. occidentalis*) and were comparable to the Fucalean family Sargassaceae based on their sympodial bifurcating patterns (Silberfeld et al. 2010). Since it was ambiguous to assign these extinct fossil genera to any extant genus, and the origin of Sargassaceae must predate the origin of the earliest fossil assigned to the family, the mid‐point of the Miocene deposits at 15 mya was set as a hard minimum bound for the stem node of Sargassaceae. A lognormal distribution (mean ± SE = 2.0 ± 0.85) with 95% highest posterior density (HPD) spanning 16.8 to 44.9 mya (minimum bound + 30 Ma; Wood et al. 2013) was applied to calibrate the node.

Eight independent Monte Carlo Markov Chain (MCMC; Altekar et al. 2004) runs were performed using BEAST 2.4.6 on the molecular data with fossil constraints, implemented at the Cyberinfrastructure for Phylogenetic Research Science Gateway 3.3 (Miller et al. 2010). Markov chains were run for 60 million generations and sampled every 1,000th generation. Convergence of all parameters was monitored using Tracer 1.6 (Rambaut et al. 2014), and a burn‐in of 10 million generations was determined. The post‐burn‐in output tree files from the eight runs were combined using LogCombiner 2.4.7 (Bouckaert et al. 2014), resampled at a lower frequency of one per 20,000 trees, and summarized as a maximum clade credibility (MCC) chronogram with TreeAnnotator 2.4.7 based on 20,000 posterior trees.

### Ancestral range estimation

The maximum likelihood‐based dispersal‐extinction‐cladogenesis (DEC) biogeographic model (Ree and Smith 2008) was applied to infer the evolution of geographic ranges and estimate the dispersal and local extinction rates using R 3.4.2 (R Core Team 2013) package BioGeoBEARS (Matzke 2013). DEC accounts for subset inheritance of sister species ranges from sympatric speciation events, which other biogeographic models like DIVA and BAYAREA do not (Matzke 2013). An additional model accounting for founder‐event speciation (+*J*) in island clades was assessed and compared with the DEC model (Matzke 2013, 2014). The rare jump dispersal event was reflected in the ancestral range estimation through a genetically diverging isolated lineage. The model was fitted to the MCC tree and species distribution data, with geographic ranges demarcated according to Vieira et al. (2017) and bioregionalization of coastal areas by Spalding et al. (2007). A total of five areas were defined: Central Indo‐Pacific (A), Western Indo‐Pacific (B), Eastern Indo‐Pacific (C), Tropical Eastern Pacific (D), and Atlantic (E). Geographic ranges of the 99 *Sargassum* species were obtained from relevant publications (Tseng et al. 1985, Tseng and Lu 1988, Yoshida 1988, Lee and Yoo 1992, Trono 1992, Ajisaka et al. 1994, 1995, 1999, Phillips 1995, Silva et al. 1996, Ajisaka 2002, Yoshida et al. 2002, Noiraksar and Ajisaka 2008) compiled at AlgaeBase (http://algaebase.org) and coded for these areas (Table S2 in the Supporting Information). Dispersal rates for area combinations of ocean basins were relaxed to allow for equal dispersal probabilities, and all possible dispersal directions were permitted considering the possibility of high connectivity among marine regions.

## Results

### Time‐calibrated phylogeny of *Sargassum*

**Figure 1:**
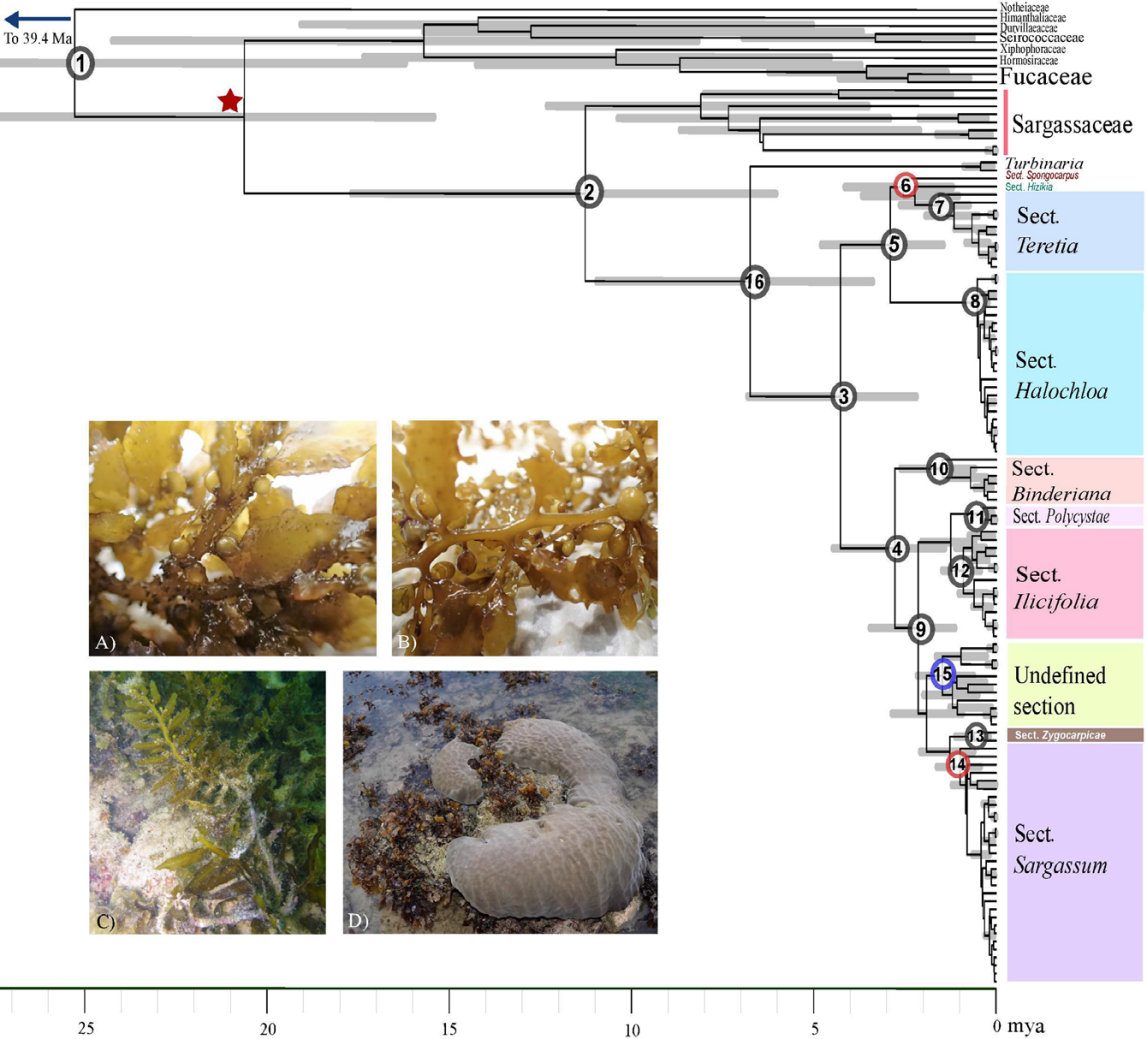
Maximum clade credibility chronogram from the BEAST analysis. Gray bars represent 95% highest posterior density (HPD) intervals of node ages. The calibrated node is denoted by a red star. Posterior probabilities of the numbered nodes (see Table 1) are denoted by color: PP > 95: black; 95 ≤ PP < 90: red; PP ≤ 90: blue. Each colored box on the right represents a section within subgenus *Sargassum*. The inset shows: A) *Sargassum polycystum*; B) *Sargassum* sp.; C) *S. swartzii* (photo credit: J.K.Y. Low); and D) coral‐*Sargassum* interaction (photo credit: J. Fong).

Following the removal of burn‐in samples, the effective sample sizes (ESS) were above 200 for all divergence time parameters. The Bayesian posterior probabilities (PP) of selected clades along with the inferred ages and 95% HPDs are presented in Table 1, with the corresponding labelled nodes shown in Figure 1. The fossil calibration point at the origin of the Sargassaceae family was estimated to have a mean age of 20.6 mya, and the origin of *Sargassum* was dated to be Late Miocene with mean age of 6.7 mya. The internal deep nodes of the *Sargassum* lineages from 4.3 to 1.6 mya representing taxonomic sections were moderately to strongly supported (PP > 0.9), while shallower nodes representing interspecific relationships were less strongly supported.

**Table 1:** Bayesian estimates for selected nodes on the chronogram in Figure 1, showing posterior probability, mean age, and 95% highest posterior probability (HPD) interval for each node of interest

Node	Description	Posterior probability	Mean age (mya)	95% HPD Interval (mya)
1	Fucales	1	25.1	16.4–39.4
2	Diversification of Sargassaceae	1	11.3	6.5–17.9
3	Diversification of *Sargassum*	1	4.3	2.2–6.8
4	Subgen. *Sargassum*	1	2.8	1.4–4.3
5	Subgen. *Bactrophycus*	1	2.9	1.5–4.8
6	–	0.93	2.6	1.3–4.1
7	Sect. *Teretia*	1	1.6	0.8–2.6
8	Sect. *Halochloa*	1	0.5	0.2–0.9
9	–	1	2.1	1.1–3.3
10	Sect. *Binderiana*	1	1.5	0.6–2.6
11	Sect. *Polycystae*	1	0.4	0.1–0.8
12	Sect. *Ilicifolia*	1	0.9	0.5–1.5
13	Sect. *Zygocarpicae*	0.99	0.6	0.2–1.1
14	Sect. *Sargassum*	0.95	1.0	0.5–1.6
15	Undefined section	0.83	1.5	0.6–2.6
16	Origin of *Sargassum*	1	6.7	3.4–11.0

*Sargassum* was recovered as sister taxon to genus *Turbinaria* with strong support (PP = 1.00). The divergence of *Sargassum* into two clades – subgenera *Sargassum* (PP = 1.00) and *Batrophycus* (PP = 1.00) – was estimated at 4.3 mya in the early Pliocene before closure of the Isthmus of Panama. Subsequent diverging lineages at subgenus level occurred throughout the Pleistocene, with the earliest diverging section *Spongocarpus* at 2.6 mya and the most recent section *Polycystae* at 0.4 mya (Fig. 1).

### Biogeographic analysis

**Figure 2:**
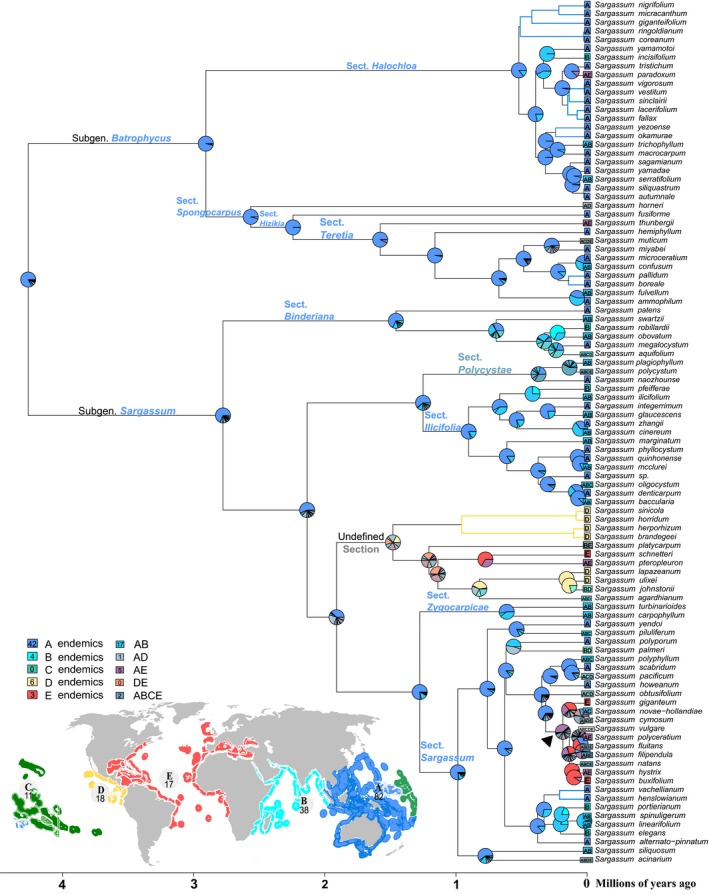
Ancestral range estimation of *Sargassum* species under the DEC + *J* model fitted onto a time‐calibrated phylogeny (Fig. 1). Tip symbols represent contemporary geographic ranges of extant taxa, and the nodal pie diagrams reflect ancestral ranges of the common ancestor. Colored taxonomic groups correspond to their ancestral range of the highest probability, and colored branches indicate maximal probability of single area distribution. Marine areas used in the analysis are Central Indo‐Pacific (A), Western Indo‐Pacific (B), Eastern Indo‐Pacific (C), Tropical Eastern Pacific (D), and Atlantic (E).

Richness comparisons based on species included in the phylogenetic analysis revealed that the Central Indo‐Pacific region (A) has the highest species richness with 82 spp., while its neighboring region (B) harbors fewer species (38 spp.). The large area of Central Indo‐Pacific combined with the Indian Ocean (AB) has the highest species richness (17 spp.) in comparison with other area combinations. The Tropical Eastern Pacific (D) has similar species richness (18 spp.) as the Atlantic (E; 17 spp.), while the Eastern Indo‐Pacific (C) has 11 species (Fig. 2). When all 360 extant *Sargassum* species were considered, we found records of 277 species in the CIP (A) and 111 species in region B. Regions C, D, and E have lower species richness of 12, 35, and 42, respectively.

The DEC model (AIC = 540.3; log‐likelihood = ‐267.5) was identified as being as well fit as the DEC + *J* model (AIC = 539.1; log‐likelihood = −267.1; *p *=* *0.38) when the five marine areas were analyzed under relaxed constraints for region combinations. The model recovered an unequivocal emphasis placed on stochastic dispersal in the range evolution of species (*d *=* *0.281 > *e *=* *0 > *j *=* *0).

Specifically, the model supported a Central Indo‐Pacific origination of the *Sargassum* ancestor, as well as for ancestors of subgenera *Sargassum* and *Batrophycus* (Fig. 2). Ancestors of the sections within the *Sargassum* subgenera shared the same ancestral area in the Central Indo‐Pacific, except for section *Polycystae* – with widespread ancestral range and present globally except in the Tropical Eastern Pacific – and the undefined section whose most recent common ancestor could be from the Central Indo‐Pacific or Tropical Eastern Pacific (Fig. 2). The common ancestor of the subclade (▲ in Fig. 2) within section *Sargassum* ranged across both the Atlantic and Pacific, with the majority of their descendant taxa occupying both the Pacific and Atlantic Ocean basins.

Overall, results lent support for a Central Indo‐Pacific origination and initial diversification of the *Sargassum* genus. Lineages began dispersing into the Atlantic less than 1.5 mya in two clades independently (undefined section and subclade ▲ in Fig. 2) and among five phylogenetically disparate species while maintaining their Pacific distribution.

## Discussion

The study of historical biogeographic patterns requires a comprehensive phylogeny that integrates clade age and geographic range data (Moore and Donoghue 2007). With recent advancements in DNA sequencing techniques, molecular analyses have been used to complement morphological analyses for a clearer understanding of the evolutionary relationships among *Sargassum* sections and species (Mattio and Payri 2011). Thus, the focus of the last decade has been to reconstruct molecular phylogenies that are not fossil‐calibrated and thus not scaled to geological time (Cho et al. 2012). For the first time, we use three commonly sequenced markers and a stem species of Sargassaceae to infer a time‐calibrated phylogeny focusing on the *Sargassum* genus and, furthermore, produce a set of Bayesian posterior trees for capturing the uncertainties of the tree topology and branch lengths.

The phylogeny recovered here is broadly congruent with the reconstruction in Dixon et al. (2014), which focused on the revision of the subgenera *Batrophycus* and *Sargassum* using molecular sequences of *ITS‐2*,* cox3,* and *rbc*LS. Two major clades representing the subgenera *Batrophycus* and *Sargassum* diverge into clades corresponding to the taxonomic sections (Fig. 1). Furthermore, in reconstructing the phylogeny of the brown algal crown radiation (BACR) group, Silberfeld et al. (2010) also included two *Sargassum* species and estimated the age of *Sargassum*, obtaining a 95% HPD lower bound for the divergence between the two species at 5 mya, which overlaps with the 95% HPD for the age of the diversification inferred here (2.2–6.8 mya). The origination of *Sargassum*, however, is estimated to be more recent in the present study (95% HPD: 3.4–11.0 mya) compared to the estimate of 22 mya as the lower bound of the 95% HPD by Silberfeld et al. (2010). The difference in time estimation for the origin of *Sargassum* could be attributed to the inclusion here of a closely related outgroup *Turbinaria* – not present in the previous study – that causes the descendant nodes (from node 3 in Fig. 1) to be pushed forward in time.

Biogeographic events and fossil records can both be incorporated in time‐calibrated phylogenetic analyses. For example, the formation of the Isthmus of Panama at 3.1 mya (Cowman and Bellwood 2013) can be used to calibrate rates of molecular evolution by applying the time of divergence of populations isolated by the rising land barrier (Knowlton et al. 1993, Knowlton and Weigt 1998, Lessios 2008). Such biogeographic events are essential in driving global marine reorganization and in creating distinct habitats for isolated populations, which then evolve on different trajectories (Lessios 2008). However, we find no reciprocally monophyletic sister clades of Atlantic and Pacific *Sargassum* species available for dating the origin of Atlantic and Pacific lineages, indicating limited differentiation between ocean realms. Furthermore, this vicariance event appears to be much older than the origin of all the Atlantic *Sargassum* lineages. Taken together, these phylogenetic results suggest that the dispersal between Pacific and Atlantic Oceans occurred much later after the closure of the Central American Seaway and thus have not been used to calibrate the phylogeny. Fossil records were instead applied to date the origin of Sargassaceae. Uncertainties concerning the age of the fossil were incorporated by specifying a loose maximum bound to avoid overconfidence in the calibration point (Parham et al. 2012). Due to the limited fossil data available for Phaeophyceae (Silberfeld et al. 2010), uncertainties in the origination and diversification dates of *Sargassum* span several million years (5–7 mya; Table 1).

High diversity regions differ for every marine taxon. However, there are marine taxa with largely concordant diversity hotspots, particularly if they live in the same habitats such as coral reefs (Bellwood and Meyer 2009). A more precise understanding of coral reef biogeography can be achieved by integrating not just coral and fish distributions, which are well studied, but also macroalgal diversity to test the factors driving diversification on reefs (Etti and Schils 2016). Here, ancestral range estimation has revealed the origin of *Sargassum* to be in the Central Indo‐Pacific (CIP) region with high certainty. From the CIP, *Sargassum* experienced few and recent dispersal events to the Atlantic in the last 1.5 mya, long after the closure of the Central American Seaway. The macroalgae *Lobophora* and *Portieria* show a parallel biogeographic history, originating in the CIP with high diversification within the region, followed by subsequent independent dispersal events into other marine realms (Vieira et al. 2017, Leliaert et al. 2018).

Similarly, for *Sargassum*, there was a profusion of early speciation events clearly reconstructed to be occurring within the CIP. This region represents the ancestral range of both subgenera *Bactrophycus* and *Sargassum* as well as most of their constituent sections (except *Polycystae* and the undefined section; Fig. 2). The abundance of speciation events in the CIP is likely conservative because it is the most poorly sampled of the five analyzed areas, whereas species from areas C, D, and E (Eastern Indo‐Pacific, Tropical Eastern Pacific, and Atlantic, respectively) are more than 40% sampled on our phylogeny. Clearly, prolific speciation has resulted in high species richness in the CIP, a pattern bearing stark similarity to that of stony corals (Veron et al. 2015). However, maintenance of the richness gradient for corals appears to be driven by range expansion of species into the CIP (Huang et al. 2018), whereas for *Sargassum*, cladogenesis in the CIP has maintained the high species richness in the region.

The CIP had undergone many climatic fluctuations in the Pleistocene (Pillans et al. 1998). In particular, the drying and subsequent re‐colonization of marine habitats during glacial–interglacial periods of both the Sunda and Sahul shelves created opportunities for the exploitation of new niches (Hofreiter and Stewart 2009, Bowen et al. 2013). Repeated, successive isolation and mixing of populations caused by the fluctuating sea level have long been recognized to promote speciation (Hallam 1985), which in the case of *Sargassum* coincided with the time of most of its speciation events. Despite the complexity of marine algal biogeography with each taxon exhibiting a distinct diversity pattern (Etti and Schils 2016), the locality of origin for many brown algal (Phaeophyceae) genera appears comparable. Here, the ancestral range of *Sargassum* is estimated to be in the CIP (Fig. 2). Similarly, the ancestral range of an older brown alga *Lobophora*, which originated in the Cretaceous period, is in the Indo‐Pacific region (Vieira et al. 2017). Younger brown algae that evolved in the early Cenozoic era, such as *Fucus*, also have their origination placed in the Pacific (Cánovas et al. 2011).

There have been past speculations on the ancestral ranges of certain taxonomic groups within *Sargassum* based exclusively on analyzing the species richness, endemism, and oceanic currents among the marine realms. For instance, Phillips (1995) hypothesized that the ancestral area of subgenus *Sargassum* is possibly in the vicinity of Baja California in the Tropical Eastern Pacific (TEP), evident from the high degree of local endemism and considerable species richness. However, species ranges are dynamic and can change over time (Jablonski et al. 2013). Endemism could also be the result of range contraction, which is associated with paleo‐endemics instead of newly evolved species (Bellwood and Meyer 2009). To account for these dynamics, we use a robust time‐calibrated phylogeny and species range data to infer the CIP origin of subgenus *Sargassum* with higher certainty than previous studies (Fig. 2).

The subclade within section *Sargassum* (labelled ▲ in Fig. 2) is inferred to have a joint ancestral range of CIP and Atlantic with relatively high level of certainty. This raises an intriguing hypothesis about its biogeographic history. The relatively recent split of the clade into CIP and Atlantic species (approximately 0.2–0.4 mya) suggests a dispersal into the Atlantic long after the formation of the Isthmus of Panama. A possible route would be across the Pacific Ocean and around the coast of South America via the Antarctic Circumpolar Current (ACC), which has been known to disperse floating macroalgae like *Macrocystis* and *Durvillaea* around the coasts of the Subantarctic (Dixon et al. 2014, Hawes et al. 2017). In other words, the ACC may have driven the dispersal of *Sargassum* – possibly species with temperate distributions (Yamasaki et al. 2014) – into the Atlantic via the Drake Passage (Dixon et al. 2014, Hawes et al. 2017). A more comprehensive phylogeny including all members of the section *Sargassum*, and incorporating past climate and ocean circulation patterns, is required to test this biogeographic hypothesis more precisely.

Studies utilizing published species records and sequence data are subject to limitations such as inaccurate species identities tagged to the data, which could result in incorrect range estimations and even bias the biogeographic inferences (Costa et al. 2015). However, given that the biogeographic range categories utilized here encompass large marine regions, small uncertainties of individual species records at range boundaries are unlikely to affect the analysis. Furthermore, we have taken steps to curate the species nomenclature and geographic data rigorously, and minimized instances of species misidentification by using only sequence data from algal taxonomists and other carefully vetted sources (datasets and species information available at Zenodo; https://doi.org/10.5281/zenodo.3403402; Tables [Link], [Link], [Link] in the Supporting Information).

## Conclusions

This study shows that high *Sargassum* richness in the Central Indo‐Pacific region is attributable to diversification within the CIP, rather than dispersal from peripheral regions into the CIP. Range evolution into other marine realms appears to have been driven primarily by dispersal. Future studies should focus on more complete taxon sampling and apply more gene markers to place most, if not all, *Sargassum* species on the phylogeny in order to produce more precise divergence time and ancestral range estimates. Past dispersal routes could also be traced by reconstructing paleoclimatic and paleoceanographic changes. Understanding the biogeographic history of *Sargassum* will pave the way for projecting range evolution of the macroalgal genus into the future, which is pivotal for developing conservation strategies in the event of climate change‐induced macroalgal encroachment into coral reef habitats.

We thank J.K.Y. Low and J. Fong for providing field photographs and are grateful to F. Leliaert and two anonymous reviewers for constructive reviews. This research is supported by the National Research Foundation, Prime Minister's Office, Singapore under its Marine Science R&D Program (MSRDP‐P03; R‐154‐000‐A25‐281).

## Conflict of Interest

The authors declare that they have no conflict of interest, and this article does not contain any studies with animals performed by any of the authors.

## Supporting information


**Table S1**. GenBank accession numbers of sequences used in the analyses.

**Table S2**. Locality data of five marine realms; where 1 represents presence and 0 absence: A) Central Indo‐Pacific, B) Western Indo‐Pacific, C) Eastern Indo‐Pacific, D) Tropical Eastern Pacific, and E) Atlantic.

**Table S3**. Geographic records for each species, adapted from AlgaeBase.

**Table S4**. Sources of the sequences used in this study and their respective voucher specimens and publication information.

**Table S5**. Sources of species taxonomy, adapted from AlgaeBase.
